# Pediatric Atlanto-Occipital Assimilation With Dynamic Instability: A Case of Successful Occipitocervical Fusion

**DOI:** 10.7759/cureus.109849

**Published:** 2026-05-28

**Authors:** Tomo Kinoshita, Vijay M Ravindra, Paul Porensky, Michael Lawless

**Affiliations:** 1 Neurosurgery, U.S. Naval Hospital Okinawa, Okinawa, JPN; 2 Neurosurgery, Naval Medical Center San Diego, San Diego, USA; 3 Neurological Surgery, MaineHealth Neurosurgery and Spine, Scarborough, USA

**Keywords:** atlanto-occipital assimilation, case report, clivo-axial angle, craniovertebral junction, dynamic instability, occipitocervical fusion, pediatric spine

## Abstract

Atlanto-occipital assimilation (AOA), also known as atlas occipitalization, is a rare congenital craniovertebral junction anomaly characterized by partial or complete fusion of the atlas with the occipital bone. It often remains asymptomatic until adulthood, and presentation in childhood is rare.

We report a nine-year-old boy with partial AOA who developed early-onset neurological symptoms. He presented with a one-year history of intermittent neck pain and transient right-hand clumsiness and numbness, which were provoked by neck extension. Imaging demonstrated a C1 hemiring, partial right-sided AOA, mild basilar invagination with the odontoid tip extending 6 mm above Chamberlain’s line, mild occiput-C1 instability, and dynamic C1-C2 instability. MRI showed ventral cervicomedullary indentation without associated medullary signal abnormality, and the clivo-axial angle (CXA) was reduced to approximately 117°. No other associated congenital anomalies were identified. Given the early neurological involvement, dynamic instability, and pathological clivo-axial angulation, occipitocervical fusion was performed using an occipital plate, C2 pedicle screws, and an autologous rib graft, without significant donor-site morbidity. The patient recovered uneventfully, with complete symptom resolution and preserved functional neck mobility. Six-month follow-up imaging confirmed stable instrumentation.

This case highlights that neurological symptoms may occur in children with AOA even without marked static cord compression when dynamic instability and pathological clivo-axial angulation are present. Early stabilization may help prevent irreversible neurological deterioration in carefully selected pediatric patients.

## Introduction

Atlanto-occipital assimilation (AOA), also known as atlas occipitalization, is a congenital anomaly characterized by failure of segmentation between the atlas and the occipital bone, resulting in partial or complete fusion of the atlas and occiput [1,2]. The craniovertebral junction represents a complex transition zone between the mobile cranium and the cervical spine, where structural abnormalities may directly affect adjacent neural and vascular structures, including the brainstem and upper cervical spinal cord [3,4]. This anomaly results from failure of normal segmentation between the fourth occipital and the first three cervical somites beginning in the fourth fetal week, and an association with mutations of Hox gene homologues has been suggested in mouse models [5].

AOA is relatively rare, with a recent systematic review estimating its prevalence to be approximately 0.64% in the general population [6]. AOA may be associated with basilar invagination, hypoplastic condyles, and fusion with adjacent vertebral bodies [2,6]. It is also frequently associated with other craniovertebral junction anomalies, including Chiari malformation and atlantoaxial instability, which may further contribute to neurological compromise [7]. The clinical presentation varies widely, ranging from incidental radiographic findings to progressive neurological deficits, depending on the degree of compression and associated anatomical abnormality [8]. Although many patients remain asymptomatic for prolonged periods, clinical features typically appear after the third decade of life [9]. Symptomatic cases may present with neck pain, myelopathy, sensory disturbance, vertebrobasilar insufficiency, or progressive neurological dysfunction due to chronic mechanical compression or vascular compromise [9,10].

Pediatric presentation is uncommon, possibly because the elasticity of the developing ligaments and flexibility of the immature spine may partially compensate for craniocervical instability, delaying neurological symptoms until later in life as spinal flexibility decreases [8]. Therefore, early neurological symptoms in pediatric patients may suggest clinically significant dynamic instability even when static anatomical deformity appears relatively mild on imaging. We report a rare pediatric case of symptomatic AOA with dynamic instability requiring occipitocervical fusion.

## Case presentation

A nine-year-old boy presented with a one-year history of intermittent popping sensation in the neck during extension, occasional headaches, and brief episodes of right-hand numbness. The right-hand numbness and clumsiness were provoked by neck extension during self-manipulation of the neck. Cervical radiographs suggested a congenital upper cervical anomaly. Neurological examination showed no fixed motor weakness, gait disturbance, bowel or bladder dysfunction, or other long-tract signs. CT demonstrated a C1 hemiring, partial right-sided atlanto-occipital fusion, and mild basilar invagination with the odontoid projecting 6 mm above Chamberlain’s line (Figure 1). No other associated congenital anomalies or syndromic features were identified on clinical or radiographic evaluation.

**Figure 1:**
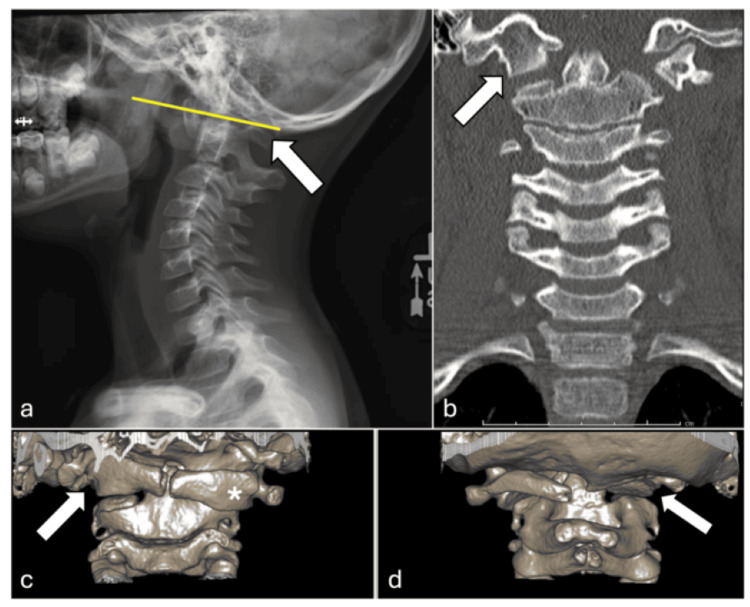
Preoperative imaging. (a) Lateral cervical radiograph demonstrating partial assimilation of the right posterior arch of C1 to the occipital bone (white arrow) and mild basilar invagination with the odontoid projecting above Chamberlain’s line (yellow line). (b) Coronal CT showing asymmetric fusion between the right posterior arch of C1 and the occipital condyle (white arrow). (c) Three-dimensional CT reconstruction (anterior view) illustrating right-sided atlanto-occipital assimilation (white arrow) and a C1 hemi-ring deformity with preservation of the anterior arch (asterisk). (d) Three-dimensional CT reconstruction (posterior view) demonstrating right-sided fusion between the posterior arch of C1 and the occipital bone (white arrow).

Dynamic imaging demonstrated mild occiput-C1 instability and dynamic C1-C2 instability, including motion-dependent widening and reduction of the atlanto-dental interval (Figure 2). Preoperative sagittal T2-weighted MRI demonstrated mild ventral indentation of the cervicomedullary junction by the odontoid process without associated medullary signal abnormality, and the clivo-axial angle (CXA) was reduced to 117° (Figure 3). Given the presence of early neurological symptoms, dynamic instability, and pathological clivo-axial angulation, surgical stabilization was recommended to reduce the risk of progressive neurological deterioration related to congenital craniocervical instability.

**Figure 2:**
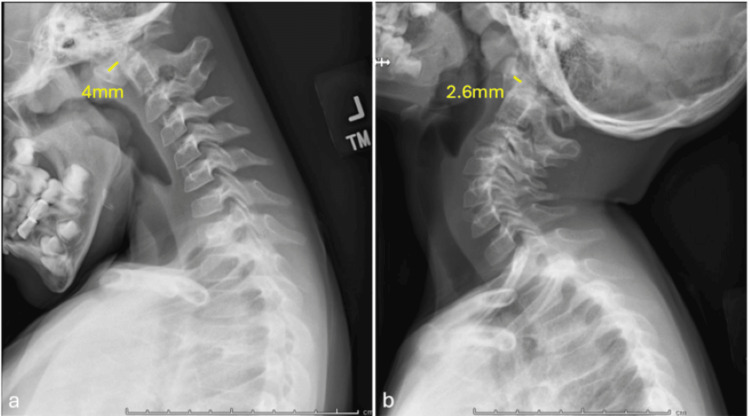
Dynamic lateral radiographs in flexion and extension views. Dynamic lateral cervical radiographs obtained in flexion (a) and extension (b) demonstrating motion-dependent widening and reduction of the atlanto-dental interval (4 mm in flexion and 2.6 mm in extension; yellow measurement lines), consistent with slight craniovertebral instability. No frank atlantoaxial subluxation is observed.

**Figure 3:**
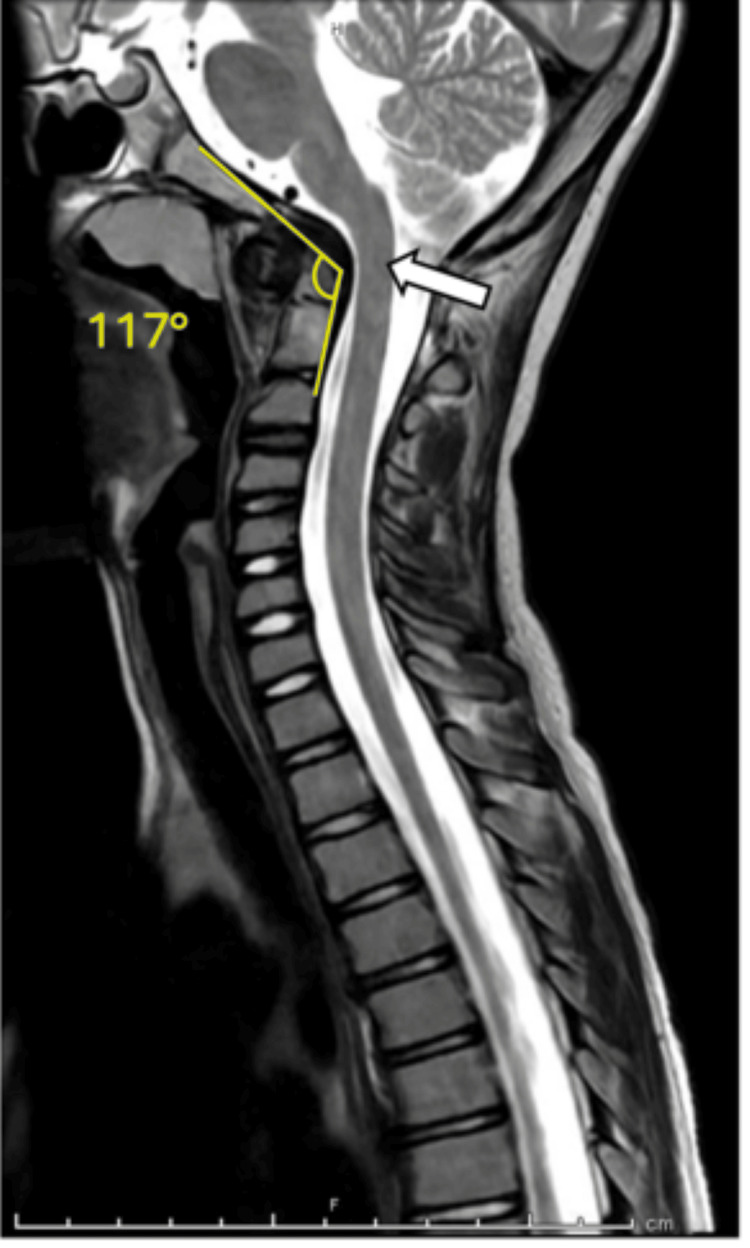
Preoperative sagittal T2-weighted MRI. Sagittal T2-weighted MRI demonstrating mild ventral indentation of the cervicomedullary junction by the odontoid process (white arrow) without medullary signal change. The clivo-axial angle was reduced to 117°.

Occipitocervical fusion was performed using an occipital plate, C2 pedicle screws, and an autologous rib graft. BMP-2, at a dose of 8.4 mg, was applied as an osteoinductive adjunct to promote arthrodesis. Preoperative imaging confirmed the absence of an aberrant vertebral artery, allowing safe C2 pedicle screw placement with image guidance. The patient was placed in a Mayfield headholder, and the neck was positioned in neutral alignment without significant traction. The autologous rib graft served as bone graft material. The postoperative course was uneventful, and the patient was discharged on postoperative day 3. No significant donor-site morbidity was observed at the rib graft harvest site. At three weeks, his preoperative symptoms had resolved completely. Radiographs at three months demonstrated stable alignment (Figure 4). At the six-month follow-up, CT confirmed stable instrumentation without evidence of osseous or hardware complications. The patient remained neurologically intact without recurrence of symptoms, maintained preserved functional neck mobility for daily activities, and was participating in regular activities without limitation.

**Figure 4:**
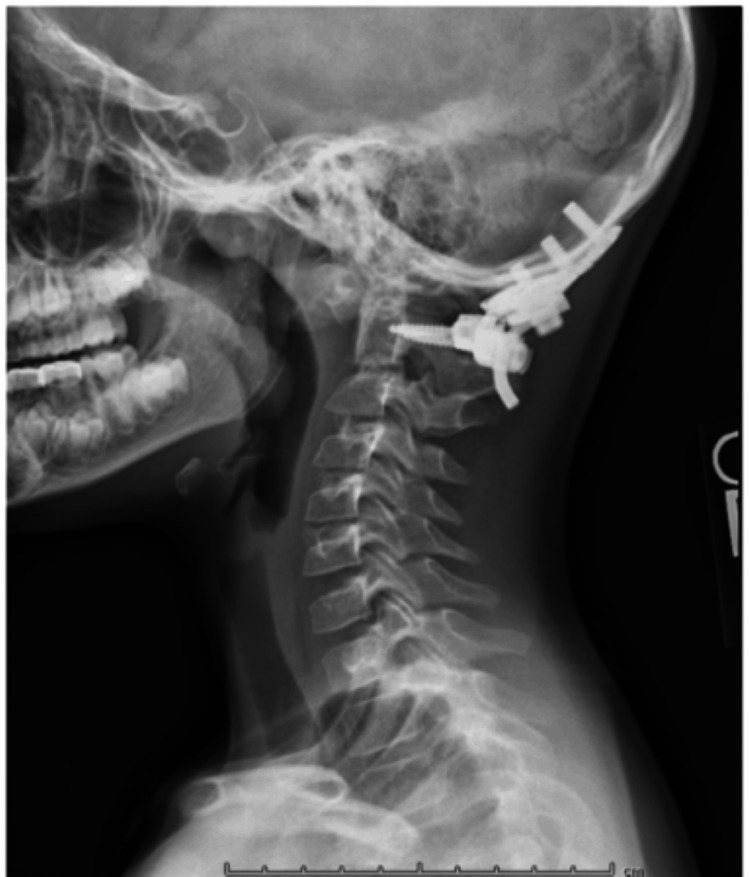
Postoperative lateral radiograph. Lateral cervical radiograph obtained at three-month follow-up demonstrating stable occipitocervical instrumentation with satisfactory alignment and no evidence of hardware failure.

## Discussion

AOA may coexist with basilar invagination or C1 anomalies [2,11]. Although often asymptomatic, instability or mechanical compression can cause neurological symptoms [7,12]. Most symptomatic cases reported in the literature involve adults presenting with progressive myelopathy, whereas pediatric patients often remain asymptomatic or develop symptoms later in life [1,9]. This delayed presentation may be partly explained by the elasticity of the developing ligaments and flexibility of the immature spine, which can partially compensate for craniocervical instability [8]. Therefore, when neurological symptoms occur early in childhood, developmental anatomical vulnerability and dynamic instability should be carefully considered.

Our case demonstrates that neurological symptoms may occur even in the absence of marked static cord compression when dynamic instability and pathological clivo-axial angulation coexist. In this patient, right-hand numbness and clumsiness were provoked by neck extension associated with self-manipulation of the neck, supporting a dynamic mechanism rather than fixed compression alone. This phenomenon may be mediated by pathological clivo-axial angulation, reflecting abnormal mechanical strain on the brainstem and upper cervical spinal cord rather than fixed direct compression alone [13]. Such a presentation in a pediatric patient with AOA appears to be infrequently reported.

Although occipitocervical fusion in pediatric patients may raise concerns regarding future spinal growth and restriction of cervical mobility, surgical stabilization was considered necessary because the risk of progressive and potentially irreversible neurological injury outweighed these limitations. Previous adult studies have emphasized the biomechanical significance of reduced clivo-axial angulation. Nagashima and Kubota reported that a CXA below 130° may produce ventral brainstem compression and suggested that the angle should be corrected to a more physiological alignment during fusion stabilization [14]. Henderson et al. also proposed that pathological clivo-axial angulation below 135°, craniocervical instability, or ventral brainstem compression may warrant consideration of occipitocervical reduction, fusion, and stabilization in symptomatic patients [13]. Although these data are derived primarily from adult patients and may not be directly generalizable to pediatric cases, the present patient developed neurological symptoms at nine years of age despite the compensatory flexibility of the pediatric spine. Given that symptomatic AOA may be associated with progressive neurological deterioration over time, the markedly reduced CXA of 117°, dynamic instability, and ventral cervicomedullary indentation supported the decision to proceed with surgical stabilization. In this case, the risk of progressive and potentially irreversible neurological injury was considered to outweigh the expected limitations in cervical mobility and potential effects on future spinal growth.

Consistent with this rationale, rigid occipitocervical fusion is generally recommended for symptomatic or unstable cases [5,9]. In pediatric patients, careful surgical planning is particularly important because rigid fixation must achieve stability while minimizing malalignment and avoiding neurovascular injury. Prior studies have suggested favorable neurological outcomes following occipitocervical stabilization in pediatric patients with craniovertebral junction anomalies [15,16]. Pediatric patients typically recover well, but long-term follow-up is essential because spinal growth, cervical alignment, fusion maturation, and functional range of motion may evolve over time [17]. In the present case, early clinical recovery and stable six-month imaging findings were encouraging; however, continued follow-up remains necessary to assess fusion maturation, cervical alignment, and functional range of motion during growth. These considerations support timely stabilization in carefully selected pediatric patients with symptomatic instability.

Clinicians should maintain a high index of suspicion for AOA and dynamic craniocervical instability in pediatric patients presenting with mechanical neck symptoms and transient neurological signs, even in the absence of marked static cord compression.

This report is limited by its single-case nature, and further studies are needed to clarify the optimal management of similar pediatric cases.

## Conclusions

Pediatric AOA is rare but may present with early neurological symptoms despite the increased flexibility of the developing spine. In children with mechanical neck symptoms or transient neurological signs, careful radiological evaluation, including assessment of dynamic instability and the CXA, is important for identifying clinically significant craniocervical instability. Timely surgical stabilization may help prevent irreversible neurological deterioration in selected pediatric patients.
